# The mental health impact of climate change on Pacific Islanders: A systematic review focused on sea level rise and extreme weather events

**DOI:** 10.1177/10398562241312865

**Published:** 2025-01-03

**Authors:** Netsanet Ayele Mengesha, Zoltan Sarnyai

**Affiliations:** Laboratory of Psychiatric Neuroscience, Australian Institute of Tropical Health and Medicine, 8001 James Cook University, Townsville, QLD, Australia

**Keywords:** climate change, mental health, sea level rise, pacific islands, mental disorder

## Abstract

**Objective:**

This systematic review investigates the impact of climate change on the mental health of Pacific Island Nations (PINs), with a focus on identifying culturally tailored interventions and appropriate research methodologies to address these impacts.

**Method:**

A systematic review of peer-reviewed literature up to May 18, 2024, was conducted using the Preferred Reporting Items for Systematic Review and Meta-Analysis (PRISMA) protocol and the Population, Interest Area, and Context (PICo) framework. Empirical studies on the impact of climate change on mental health in PINs were evaluated by using the Quality Assessment Tool for Studies with Diverse Designs (QATSDD).

**Results:**

Six studies from the Solomon Islands, Cook Islands, and Tuvalu were identified, indicating significant mental health impacts from sea level rise (SLR) and extreme weather events with compounding and mitigating effects across diverse groups. The Skills for Life Adjustment and Resilience (SOLAR) program was highlighted as a promising culturally adapted intervention.

**Conclusion:**

Climate change significantly impacts mental health, particularly in PIN communities facing SLR and Extreme Weather Events (EWE). Culturally sensitive interventions, local knowledge, and further research are vital to mitigate these effects and support well-being.

Since 1970, global sea levels have been rising at an accelerating rate.^1,2^ Research has shown that the primary driver of Sea Level Rise (SLR) is anthropogenic carbon dioxide (CO_2_) emissions.^3,4^ Moreover, the geological records from the past three ice age cycles (Paleogene, Neogene, and Quaternary geological periods) indicated a strong positive correlation between atmospheric CO_2_ levels and SLR.^1^ Over the past century, the rate of SLR has increased significantly, from 1.4 mm per year (between 1901 and 1990) to 3.6 mm per year between (2006-2015),^3^ with future projections suggesting a rise of 0.3 to 2 m by 2100.^5,6^ Pacific Island Nations (PINs) are especially vulnerable to these changes^7^ with regional leaders raising concerns about SLR as early as 2014.^8^ In addition to SLR, PINs also face other severe climate change related Extreme Weather Events(EWE) including cyclone, flooding, storm wave, erosion and submersion of land, coral bleaching, and droughts.^9–11^ These events can force people to involuntarily migrate or relocate,^3^ cause financial hardship, loss of cultural heritage including indigenous knowledge, and lead to a loss of community and belonging.^12^ Such challenges negatively affect individuals’ mental health.^13^ It is well documented that extreme weather events related to climate change are strongly linked to mental health impact.^14–16^ This systematic review seeks to address the question of how climate change induced SLR and EWE impact the mental health and well-being of PINs populations. It will also explore culturally appropriate interventions to mitigate mental health risks and assess the research methodologies applied in PINs contexts to ensure culturally sensitive insights.

## Methods

A systematic review of peer-reviewed literature published up to May 18, 2024, was conducted following the Preferred Reporting Items for Systematic Review and Meta-Analysis (PRISMA) protocol.^17^ The review was structured using the Population (P) = PINs, Interest Area (I) = climate change induced SLR and EWE, Context (Co) = mental health and well-being of PINs (PICo) framework^18^ to address the systematic review question. Due to the limited available data conducting a meta-analysis or meta-aggregation is not feasible. The literature was examined from seven databases: CINAHL, Emcare on Ovid, Medline Ovid, PsycINFO, Scopus, Web of Science, and Google Scholar, using the search terms related to climate change, mental health, sea level rise, Pacific Islands, mental disorder, and their synonyms. The detailed search strategy is available in Supplemental File 1. The selection of relevant research papers was based on predefined inclusion and exclusion criteria as shown on Table 1. To enhance efficiency in the literature identification process, EndNote version 20.4.1 was utilized to automatically screen and exclude articles that did not meet the inclusion criteria and duplicates. Consequently, a total of *n* = 19 duplicates removed and *n* = 11 records were identified as ineligible by the automation tool. The primary findings underwent additional screening.

**Table 1.** Inclusion and exclusion criteria

Inclusion criteria	Exclusion criteria
1. Empirical peer reviewed studies	1. All non-empirical studies including commentaries, editorial, letters, note, book sections, viewpoints, reviews, case studies, protocols
2. Considered adult and/or children’s mental health impact	2. Studies that focused on all countries except PINs
3. Focused on specifically on PINs	
4. Study designs include interventional studies, mixed studies, qualitative studies, quantitative studies	

The authors independently screened the titles and abstracts, and any conflicts were resolved by discussion. Full-text studies were screened independently by authors, with a random double check of 20% conducted for accuracy. Only empirical studies that specifically explored the impact of climate change on the mental health in PINs were chosen for critical evaluation.

Risk assessment and quality evaluation of these largely observational and mixed-methods studies were performed using the Quality Assessment Tool for Studies with Diverse Designs (QATSDD).^19^ A data extraction form was utilized in Microsoft Word as a table format (refer to Table2), capturing information about the author, study location, study period, final sample size, study design, population of interest, mental health measures, results, coping strategies, how the confounding factors controlled, comments, and References of the studies.

**Table 2.** Details of the studies covered in this systematic review

Authors	Study location	Study period	Final sample size	Study design	Population of interest	Mental health measures	Results	Coping strategies	How the confounding factors controlled	Comments	References
Asugeni et al. (2015)	Solomon Islands	Does not state the exact study period for this research	57	A cross-sectional study	Six remote village residents, that is, Kwai, Ngongosilia, Fouoge, Ou, Abitona, Canaan	Used a short survey and interviews to assess SLR’s impacts of on mental health	High worry about SLR affected mental health of individuals and community well-being	Seeking help from local and government authorities	Surveys directly asked participants if their worry was linked to SLR	The study lacks details on validating mental health measurement tools	16
				Mixed method							
Gibson et al. (2019)	Tuvalu	During 3 weeks of fieldwork on Funafuti atoll in 2015	39	Qualitative	Adult 16 key informants and 23 lay residents	Researchers compared DSM-5 psychiatric disorders with the residents’ experiences of stress and distress	Stress and distress manifest as worry, tiredness, disrupted sleep, a distracted mind, and high blood pressure	Religious beliefs and community support	Used maximal variation purposive sampling	Results may not apply to all of Tuvalu; study focused on Funafuti	17
Gibson et al. (2020)	Tuvalu	Between August and October 2016	100	Cross-sectional design using mixed-methods	General population of Funafuti atoll Tuvalu	HSCL-25 Tuvalu	Climate change stressor caused extreme distress, including sadness, anxiety, and anger	Not explored in this study	The structured interviews helped to minimize confounding factors	Systematic sampling, not random, may limit findings generalizability to Tuvaluan population	9
Gibson et al. (2021)	Tuvalu	October–November 2018	99	A quasi-experimental, control design	Adults aged 18 + from Funafuti and Nui, mild mental impairment post cyclone Pam	HSCL-25 Tuvalu, PCL-5, TIC, and PSYCHLOPS used for mental health assessment before and after SOLAR intervention	SOLAR intervention improved mental health, proving acceptable, feasible, safe, and effective for post-disaster Tuvalu	SOLAR program builds resilience and aids coping with trauma effects	ANCOVA analyses used to minimize the influence of potential confounding variables	Randomized trials needed for stronger efficacy evidence despite baseline control	18
Furusawa et al. (2021)	Solomon Islands	December 2017 (Taro and Sasamungga communities) and July 2018 (Manuopo communities)	384	Cross-sectional study	All adults in the 3 communities : Taro Island, Manuopo, and Sasamungga (control group)	HESPER (PSQ4D)	Manuopo:60.6% reported depression vs. Sasamungga 32.4%, Taro 33.9%	Not explored in this study	Logistic regression analysis was conducted to minimize risk factors	Over 30% reported depression in all three communities including control group; the study didn’t explore causes. Further research needed	8
Clissold et al. (2023)	Cook Islands	October and November 2020	11	Qualitative study design	Adults 43–56 y/o, average 55 y/o, in Cook Islands , reliant on community, agriculture, fishing, and other income streams	Content analysis grouped emotions related to drought and cyclone into themes	Extreme weather events caused emotional impacts like stress, fear, tiredness, and anger	Community cohesion, resource sharing, and reliance on indigenous knowledge	Participants asked directly about: experiences with droughts and cyclones over the past 20 years, their responses and coping strategies	Small sample and geographical bias, with most participants from the Southern group	4

## Results

**Figure 1.**
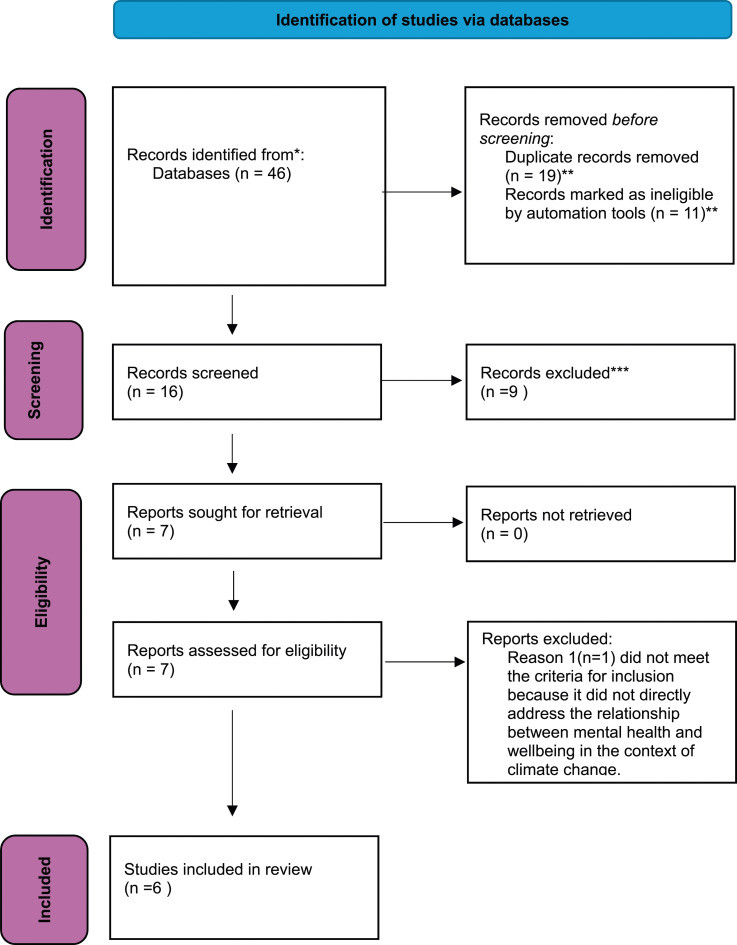
Prisma flow chart. * Number of records identified from each database from CINAHL (*n* = 0), Emcare on Ovid (*n* = 7), Medline Ovid (*n* = 4), PsycINFO (*n* = 1), Scopus (*n* = 21), web of science (*n* = 11), Google Scholar (*n* = 2). ** EndNote 20.4.1 software was utilized to streamline the selection process by identifying and excluding non-empirical studies, such as books, editorials, commentaries etc. As a result, 11 records were excluded using the automation tools provided by EndNote. Additionally, *n* = 19 duplicate records were identified and removed. *** 9 records were excluded by a human after full text screening not meeting predefined inclusion criteria.

The key search terms generated a total of 46 records as visually presented in a PRISMA^17^ flow chart (Figure 1).

After removing 19 duplicates, title and abstracts were screened of 27 studies that yielded 16 articles to apply the inclusion and exclusion criteria to. Finally, 6 studies were found eligible for quality assessment.

Four studies provided numerical data for data extraction; the other two studies were qualitative in nature. Results of the QATSDD analysis indicate that the quality of the papers is good, with two papers scoring from 62 to 71%, 3 papers scoring from 83 to 88%, 1 paper scoring 93% (Supplemental File 2). Of the 6 papers selected for extraction, two were from Solomon Islands,^20,21^ three from Tuvalu,^22–24^ and one from the Cook Islands.^25^ Each study used different methodologies and measurements to assess the mental health outcomes associated with climate change.

The studies reviewed explore the mental health impacts of climate change in PINs. Asugeni et al. (2015) and Furusawa et al. (2021) reported high levels of stress, worry, and depression linked to rising sea levels in the Solomon Islands. Gibson et al (2019, 2020, and 2021) highlighted climate-related distress in Tuvalu, focusing on social-environmental challenges, psychological distress, and the effectiveness of the SOLAR intervention program. Clissold et al. (2023) in the Cook Islands emphasized emotional impacts of extreme weather, with community and indigenous knowledge aiding resilience. These studies underscore the importance of culturally relevant mental health strategies in climate-vulnerable regions. A detailed summary of the characteristics of each paper is summarized in Table 2 as a data extraction table.

## Discussion

From the six reviewed studies, we identified four key themes including^1^ Mental health impacts of climate change by geographical location^2^; Resilience and culturally appropriate intervention^3^; Multiple stressors versus single stressor for risk of depression; and^4^ possible vicarious traumatization experience. These themes explore the mental health implications of climate change induced SLR and extreme weather events. These events include floods, cyclones, and drought.

### Mental health impacts of climate change by geographical location

The studies reviewed reveal the profound mental health impacts of climate change, particularly in small island communities across the Solomon Islands,^20,21^ Cook Islands,^25^ and Tuvalu.^22–24^ In the Solomon Islands, Asugeni et al. conducted a cross-sectional study in Malaita, exploring the mental health issues associated with SLR in two low-lying islands and coastal mainland location.^21^ The study found that 98% of participants reported that concerns about SLR influenced their everyday thinking and behavior, impacting not only themselves but also their families and communities.^21^ In Tuvalu, qualitative research uncovered distress among women and older adults, manifesting in local idioms such as “manavase” (worry) and “mafaufau mafa” (thinking too much), which correspond with symptoms of anxiety and depression.^22^ Similarly, another study in Tuvalu linked local observable climate changes, such as declining crop yields and coastal erosion, with significant psychological distress, particularly among women and those already experiencing financial hardship.^23^ In the Cook Islands, individuals reliant on agriculture and fishing reported heightened vulnerability to mental health issues due to extreme weather events like droughts and cyclones, emphasizing the connection between resource depletion and psychological distress.^25^

### Resilience and culturally appropriate intervention

Resilience strategies and culturally appropriate interventions play a crucial role in mitigating the mental health impacts of climate change. Across these studies, community cohesion,^25^ resource sharing,^25^ religion/faith,^22^ and reliance on indigenous knowledge^25^ were identified as critical coping mechanisms. In Tuvalu, the SOLAR program was piloted as a culturally adapted, scalable psychosocial intervention designed to reduce distress following disasters.^24^ The program demonstrated significant improvements in mental health outcomes, including reductions in psychological distress, Post Traumatic Stress Disorder (PTSD) symptoms, and self-identified difficulties, with these gains maintained at a 6-month follow-up.^24^ These findings underscore the importance of developing and implementing culturally tailored interventions accepted by the community and delivered by local, non-specialist facilitators.

### Multiple stressors versus single stressors for risk of depression

A cross-sectional study conducted in the Solomon Islands by Furusawa et al.,^20^ compared depression rates across three communities such as Taro Island, Manuopo community in Reef Islands, and Sasamungga being a control group. Depression was assessed using the Primary Care Screening Questionnaire for Depression (PSQ4D), while physical, mental, and social needs were measured with the Humanitarian Emergency Settings Perceived Needs Scale (HESPER). Manuopo residents exhibited a significantly higher proportion of depression (60.6%) than those in Sasamungga (32.4%) and Taro (33.9%).

The Manuopo community faces unique challenges typical of small island environments, including limited income and livelihoods, frequent food shortages, and lack of support from others such as restricted access to essential resources and information. These compounding stressors likely exacerbate mental health vulnerabilities, suggesting that the convergence of multiple stress factors may heighten depression risk more than any single factor. Interestingly, while Taro residents experience similar concerns related to SLR and have been advised to consider relocation by the government, they reported lower depression rates than Manuopo residents, possibly due to easier access to inland resources and food supplies.

### Possible vicarious traumatization experience

Research indicates that indirect exposure to traumatic events, such as through news coverage or conversations, can trigger psychological responses similar to those experienced by individuals directly impacted.^26,27^ For instance, a study conducted in New South Wales (NSW), Australia, following the 2001–2002 bushfires, estimated a 75% of risk of developing vicarious trauma among indirectly exposed individuals in the general population.^28^ Similarly, research by Furusawa et al.^20^ in the Solomon Islands found unexpectedly high rates of elevated blood pressure among the communities of Sasamungga (26.5%), Taro (12.1%), and Manuopo (13.5%). Notably, Sasamungga, a community (with low risk of SLR) showed particularly high blood pressure results, though the study did not explain the specific factors contributing to this outcome. However, it may be possible that Sasamungga residents are experiencing vicarious trauma related to climate change, manifesting as physical symptoms like increased blood pressure rather than overt emotional distress. This aligns with findings from a study in Tuvalu by Gibson et al.,^22^ which noted that individuals who appeared visibly distressed often exhibited physical symptoms, such as high blood pressure, increased heart rate, and trouble sleeping. Emotional stress was typically only mentioned when accompanied by these physical symptoms, suggesting that in cultural contexts of the PINs, physical symptoms may serve as indicators of psychological distress.^22^ Moreover, research shows that individuals exposed to psychological stress may experience temporary blood pressure spikes that normalize once the stress is alleviated.^29^ However, those with stress associated with PTSD face a higher risk of long-term rise of blood pressure.^29^

These findings highlight the potential for similar expressions of vicarious trauma, related to climate change and SLR, within the Sasamungga community. Comprehensive studies are necessary to further investigate the relationship between indirect trauma exposure and psychological or physiological responses in climate-vulnerable populations such as PINs.

### Methodologies and limitations

The methodologies used in the reviewed studies primarily involved cross-sectional designs and a combination of qualitative and quantitative methods.^20–23,25^ While these approaches provided valuable insights into the mental health impacts of climate change, several limitations were noted. The small sample sizes and geographical biases in the studies limit the generalizability of the findings. Additionally, there was a lack of information on pre-existing mental health conditions, and the cross-sectional nature of the studies prevented the examination of long-term impacts. Importantly, none of the studies employed objective, biological measures of stress, such as biomarkers which could offer a more comprehensive understanding of the biological underpinning of climate related stress.

### Research gaps

The reviewed literature highlights significant research gaps in climate change and mental health. Future studies could benefit from more robust methodological approaches, including longitudinal designs that track mental health outcomes over time. Incorporating objective measures of stress, such as such as hair cortisol, could provide insights into longer term activation of the biological stress response^30^ or heart rate and heart rate variability which are sensitive measures of the stress-related alterations of the autonomic nervous system.^31^ For example, the study that was conducted in Solomon Islands by Furusawa et al did measure the physiological health status as well as the mental health assessments. However, the high blood pressure outcome in the Sasamungga community was not explained. Therefore, further research could offer a more comprehensive understanding of the biological underpinning of climate related stress. Addressing these gaps would not only enhance our understanding of the mental health impacts of climate change but also inform the development of more effective interventions to mitigate these impacts in vulnerable communities.

## Conclusion

The reviewed studies present compelling evidence of the substantial mental health impacts of climate change on small island communities in the Pacific region. Although, this is a brief systematic review, limited by the availability of empirical studies on PIN, it offers significant and insightful contributions to the understanding of this issue. Rising sea levels and extreme weather events exacerbate psychological distress, particularly among vulnerable populations such as women, younger individuals, and those experiencing financial hardship. Traditional knowledge and community cohesion emerge as vital coping mechanisms, highlighting the resilience of these communities.

Culturally adapted interventions, like the SOLAR program, demonstrate the potential for effective mental health support in post-disaster contexts. However, future research must address the limitations of current studies, including small sample sizes, geographical biases, pre-disaster interventions, and methodological weaknesses. By enhancing the robustness of research designs and incorporating a broader socio-political perspective, we can better understand and mitigate the mental health impacts of climate change on these vulnerable communities.

Therefore, the intersection of environmental changes and mental health in Pacific Island communities requires continued attention and action. By leveraging local knowledge and resources and developing culturally sensitive interventions, we can support the mental well-being of these communities as they navigate the challenges posed by climate change.

## Supplemental Material

**Supplemental Material -** The mental health impact of climate change on Pacific Islanders: A systematic review focused on sea level rise and extreme weather events Supplemental Material for The mental health impact of climate change on Pacific Islanders: A systematic review focused on sea level rise and extreme weather events by Netsanet Ayele Mengesha and Zoltan Sarnyai in Australasian Psychiatry.

## Appendix

### Abbreviations

TIC: Tuvalu impairment checklist
HSCL-25 Tuvalu: Culturally adapted version of the 25-item Hopkins symptom checklist
DSM-5: Diagnostic and statistical manual of mental disorders, 5th edition
PCL-5: Post traumatic stress disorder (PTSD) checklist for DSM-5
PSYCHLOPS: Psychological outcome profiles
PSQ4D: Primary care screening questionnaire for depression
HESPER: Humanitarian emergency settings perceived needs scale.
