# Supplementary material for: The mental health impact of climate change on Pacific Islanders: A systematic review focused on sea level rise and extreme weather events
Source: Australas Psychiatry. 2025 Jan 3;33(2):220–7. doi: 10.1177/10398562241312865 (PMC11982582; doi:10.1177/10398562241312865)
Supplement: Supplemental Material - The mental health impact of climate change on Pacific Islanders: A systematic review focused on sea level rise and extreme weather events [file sj-pdf-1-apy-10.1177_10398562241312865.pdf]

Supplementary File 1: _Full search strategy

Cinhal(n=0)

S7	S3 OR S4	**Expanders** - Apply equivalent subjects<br>**Search modes** - Proximity	View Results (692,737)	View Details · Edit
S6	S1 OR S2	**Expanders** - Apply equivalent subjects<br>**Search modes** - Proximity	View Results (3,970)	View Details · Edit
S5	(MH "Pacific Islanders+")	**Expanders** - Apply equivalent subjects<br>**Search modes** - Proximity	View Results (260)	View Details · Edit
S4	(MH "Mental Disorders+")	**Expanders** - Apply equivalent subjects<br>**Search modes** - Proximity	View Results (652,799)	View Details · Edit
S3	(MH "Mental Health")	**Expanders** - Apply equivalent subjects<br>**Search modes** - Proximity	View Results (63,961)	View Details · Edit
S2	(MH "Sea Level Rise")	**Expanders** - Apply equivalent subjects<br>**Search modes** - Proximity	View Results (9)	View Details · Edit
S1	(MH "Climate Change+")	**Expanders** - Apply equivalent subjects<br>**Search modes** - Proximity	View Results (3,970)	View Details · Edit

S5 AND S6 AND S7 — Select a Field (optional) — Search

AND — Select a Field (optional) — Create Alert

AND — Select a Field (optional) — Clear

Basic Search Advanced Search Search History

No results were found.

Emcare (n=7)

Search History (8) View Saved

#	Searches	Results	Type	Actions	Annotations
8	5 and 6 and 7	7	Advanced	Display Results More	
7	3 or 4	690200	Advanced	Display Results More	
6	1 or 2	12554	Advanced	Display Results More	
5	exp Pacific islands/	13593	Advanced	Display Results More	
4	exp mental disease/	628681	Advanced	Display Results More	
3	exp mental health/	121378	Advanced	Display Results More	
2	exp sea level rise/	383	Advanced	Display Results More	
1	exp climate change/	12344	Advanced	Display Results More	

Medline (n=4)

Search History (8) View Saved

#	Searches	Results	Type	Actions	Annotations
8	5 and 6 and 7	4	Advanced	Display Results More	
7	3 or 4	1543685	Advanced	Display Results More	
6	1 or 2	33541	Advanced	Display Results More	
5	exp Pacific Islands/	68561	Advanced	Display Results More	
4	exp Mental Disorders/	1497647	Advanced	Display Results More	
3	exp Mental Health/	68025	Advanced	Display Results More	
2	exp Sea Level Rise/	164	Advanced	Display Results More	
1	exp Climate Change/	33541	Advanced	Display Results More	

PsycINFO (n=1)

Set	Search	Databases	Results	Save search/alert	Other actions
S1	⊞ MAINSUBJECT.EXACT.EXPLODE("Pacific Islanders") AND (MAINSUBJECT.EXACT("Mental Health") OR MAINSUBJECT.EXACT.EXPLODE("Mental Disorders")) AND (MAINSUBJECT.EXACT.EXPLODE("Climate Change") OR MAINSUBJECT.EXACT("Extreme Weather"))	APA PsycInfo®	1	Save search/alert ▼	Other actions ▼

Scopus (n=21) data base key terms

Scopus

Documents Authors Sources

21 document results

	List name	Documents	Date created	Actions
1.	SLR searched 18.05.2024	21	18 May 2024	Edit Delete

((("Climate Changes" OR "climate change" OR "climate sensitivity" OR "climate variability" OR "Rising Sea Level" OR "Sea-Level Rise" OR "sea level rise" OR "rising sea levels") AND ("Mental Hygiene" OR "mental health" OR "Mental Disease" OR "Mental Diseases" OR "Mental Disorder" OR "Mental Illness" OR "mental care" OR "mental condition" OR "mental factor" OR "mental help" OR "mental service" OR "mental state" OR "mental status" OR "mental status schedule" OR "psychic health" OR "abnormal mental state" OR "behavior disorders" OR "diseased mental state" OR "disordered mental state" OR "disturbed mental state" OR "insanity" OR "mental abnormality" OR "mental change" OR "mental confusion" OR "mental defect" OR "mental disorder" OR "mental disorders" OR "mental disorders diagnosed in childhood" OR "mental disturbance" OR "mental illness" OR "mental illnesses" OR "mental insufficiency" OR "mental symptom" OR "mentally ill" OR "neurodevelopmental disorder" OR "neurodevelopmental disorders" OR "neuropsychiatric disease" OR "neuropsychiatric diseases" OR "neuropsychiatric disorder" OR "neuropsychiatric disorders" OR "psychiatric diagnosis" OR "psychiatric disease" OR "psychiatric diseases" OR "psychiatric disorder" OR "psychiatric disorders" OR "psychiatric illness" OR "psychiatric illnesses" OR "psychiatric symptom" OR "psychic disease" OR "psychic disorder" OR "psychic disturbance" OR "psychologic disorder" OR "psychologic disturbance" OR "psychological disorder" OR "psychological disturbance" OR "psychopathology" OR "severe mental disorder" OR "severe mental disorders") AND ("pacific Islanders" OR "Micronesian People" OR "Micronesians" OR "micronesia-polynesia" OR "Pacific Island People" OR "Oceania" OR "Pacific island" OR "Pacific islands")))

Web of science (n=11) data base key terms

☐	Name	Last Modified ↓	Type	Count	
☐	SLR 19/05/24	05-19-2024 10:29	Documents	11	Edit details

((("Climate Changes" OR "climate change" OR "climate sensitivity" OR "climate variability" OR "Rising Sea Level" OR "Sea-Level Rise" OR "sea level rise" OR "rising sea levels") AND ("Mental Hygiene" OR "mental health" OR "Mental Disease" OR "Mental Diseases" OR "Mental Disorder" OR "Mental Illness" OR "mental care" OR "mental condition" OR

"mental factor" OR "mental help" OR "mental service" OR "mental state" OR "mental status" OR "mental status schedule" OR "psychic health" OR "abnormal mental state" OR "behavior disorders" OR "diseased mental state" OR "disordered mental state" OR "disturbed mental state" OR "insanity" OR "mental abnormality" OR "mental change" OR "mental confusion" OR "mental defect" OR "mental disorder" OR "mental disorders" OR "mental disorders diagnosed in childhood" OR "mental disturbance" OR "mental illness" OR "mental illnesses" OR "mental insufficiency" OR "mental symptom" OR "mentally ill" OR "neurodevelopmental disorder" OR "neurodevelopmental disorders" OR "neuropsychiatric disease" OR "neuropsychiatric diseases" OR "neuropsychiatric disorder" OR "neuropsychiatric disorders" OR "psychiatric diagnosis" OR "psychiatric disease" OR "psychiatric diseases" OR "psychiatric disorder" OR "psychiatric disorders" OR "psychiatric illness" OR "psychiatric illnesses" OR "psychiatric symptom" OR "psychic disease" OR "psychic disorder" OR "psychic disturbance" OR "psychologic disorder" OR "psychologic disturbance" OR "psychological disorder" OR "psychological disturbance" OR "psychopathology" OR "severe mental disorder" OR "severe mental disorders") AND ("pacific Islanders" OR "Micronesian People" OR "Micronesians" OR "micronesia-polynesia" OR "Pacific Island People" OR "Oceania" OR "Pacific island" OR "Pacific islands")))

Google scholars (n=2)

Full text search : “The impact of climate change on the mental health of pacific Island nations”.

Supplementary File 2 : Results of the QATSDD analysis

Asugeni et al (2015) Mental health issues from rising sea level in a remote coastal region of the Solomon Islands: Current and future.						
	Criteria	**Rate**	0 = Not at all	1 = Very slightly	2 = Moderately	3 = Complete
1	Does this study have Explicit theoretical framework	0	No mention at all	Reference to broad theoretical basis.	Reference to a specific theoretical basis.	Does this study have Explicit statement of theoretical framework and/or constructs applied to the research.
2	Does it have Statement of aims/objectives in main body of report	3	No mention at all	General reference to aim/objective at some point in the report including abstract.	Reference to broad aims/objectives in main body of report.	Explicit statement of aims/objectives in main body of report.
3	Does it have Clear description of research setting	1	No mention at all	General description of research area and background, e.g. ‘in primary care’.	General description of research problem in the target population, e.g. ‘among GPs in primary care’.	Specific description of the research problem and target population in the context of the study, e.g. nurses and doctors from GP practices in the east midlands.
4	Does it have Evidence of sample size considered in terms of analysis	1	No mention at all	Basic explanation for choice of sample size. Evidence that size of the sample has been considered in study	Evidence of consideration of sample size in terms of saturation/information	Explicit statement of data being gathered until information

				design.	redundancy or to fit generic analytical requirements	redundancy/saturation was reached or to fit exact calculations for analytical requirements.
5	Does it have Representative sample of target group of a reasonable size	2	No statement of target group	Sample is limited but represents some of the target group or representative but very small.	Sample is somewhat diverse but not entirely representative, e.g. inclusive of all age groups, experience but only one workplace. Requires discussion of target population to determine what sample is required to be representative	S ample includes individuals to represent a cross section of the target population, considering factors such as experience, age and workplace.
6	Does it have Description of procedure for data collection	3	No mention at all	Very basic and brief outline of data collection procedure,<br>e.g. 'using a questionnaire distributed to staff'.	States each stage of data collection procedure but with limited detail, or states some stages in details but omits others.	Detailed description of each stage of the data collection procedure, including when, where and how data were gathered.
7	Does it have Rationale for choice of data collection tool(s)	2	No mention at all	Very limited explanation for choice of data collection tool(s).	Basic explanation of rationale for choice of data collection tool(s), e.g. based on use in a prior similar study.	Detailed explanation of rationale for choice of data collection tool(s), e.g. relevance to the study aims and assessments of tool quality either statistically, e.g. for reliability & validity, or relevant qualitative assessment.
8	Does it have Detailed recruitment data	3	No mention at all	Minimal recruitment data, e.g. no. of questionnaire sent<br>and no. returned.	Some recruitment information but not complete account of the recruitment process, e.g. recruitment figures but no information on strategy used.	Complete data regarding no. approached, no. recruited, attrition data where relevant, method of recruitment.
9	Does it have Statistical assessment of reliability and validity of measurement tool(s)<br>(Quantitative only)	0	No mention at all	Reliability and validity of measurement tool(s) discussed, but not statistically assessed.	Some attempt to assess reliability and validity of measurement tool(s) but insufficient, e.g. attempt to establish test–retest reliability is unsuccessful but no action is taken.	Suitable and thorough statistical assessment of reliability and validity of measurement tool(s) with reference to the quality of evidence as a result of the measures used.
10	Does it have Fit between stated research question and method of data collection<br>(Quantitative)	3	No research question stated	Method of data collection can only address some aspects of the research question.	Method of data collection can address the research question but there is a more suitable alternative that could have been used or used in addition.	Method of data collection selected is the most suitable approach to attempt answer the research question
11	Does it have Fit between stated research question and format and content of data collection tool e.g. interview schedule<br>(Qualitative)	3	No research question stated	Structure and/or content only suitable to address the research question in some aspects or superficially.	Structure & content allows for data to be gathered broadly addressing the stated research question(s) but could benefit from greater detail.	Structure & content allows for detailed data to be gathered around all relevant issues required to address the stated research question(s).
12	Does it have Fit between research question and method of analysis	2	No mention at all	Method of analysis can only address the research question basically or broadly.	Method of analysis can address the research question but there is a more suitable alternative that could have been used or used in addition to offer greater detail.	Method of analysis selected is the most suitable approach to attempt answer the research question in detail, e.g. for qualitative IPA preferable for experiences vs. content analysis to elicit frequency of occurrence of events, etc.
13	Does it have Good justification for analytical method selected	3	No mention at all	Basic explanation for choice of analytical method	Fairly detailed explanation of choice of analytical method	Detailed explanation for choice of analytical method<br>based on nature of research question(s)
14	Does it have Assessment of reliability of analytical process<br>(Qualitative only)	2	No mention at all	More than one researcher involved in the analytical process but no further reliability assessment.	Limited attempt to assess reliability, e.g. reliance on one method.	Use of a range of methods to assess reliability, e.g. triangulation, multiple researchers, varying research backgrounds
15	Does it have Evidence of user involvement in design	3	No mention at all	Use of pilot study but no involvement in planning stages of study design.	Pilot study with feedback from users informing changes to the design.	Explicit consultation with steering group or statement or formal consultation with users in planning of study design.

16	Does it have Strengths and limitations critically discussed	3	No mention at all	Very limited mention of strengths and limitations with omissions of many key issues	Discussion of some of the key strengths and weaknesses of the study but not complete	Discussion of strengths and limitations of all aspects of study including design, measures, procedure, sample & analysis.
	Total score	34/48				
		71%				

Gibson et al (2019). Distressing encounters in the context of climate change: Idioms of distress, determinants, and responses to distress in Tuvalu						
	Criteria	**Rate**	0 = Not at all	1 = Very slightly	2 = Moderately	3 = Complete
1	Does this study have Explicit theoretical framework	3	No mention at all	Reference to broad theoretical basis.	Reference to a specific theoretical basis.	Does this study have Explicit statement of theoretical framework and/or constructs applied to the research.
2	Does it have Statement of aims/objectives in main body of report	3	No mention at all	General reference to aim/objective at some point in the report including abstract.	Reference to broad aims/objectives in main body of report.	Explicit statement of aims/objectives in main body of report.
3	Does it have Clear description of research setting	3	No mention at all	General description of research area and background, e.g. 'in primary care'.	General description of research problem in the target population, e.g. 'among GPs in primary care'.	Specific description of the research problem and target population in the context of the study, e.g. nurses and doctors from GP practices in the east midlands.
4	Does it have Evidence of sample size considered in terms of analysis	3	No mention at all	Basic explanation for choice of sample size. Evidence that size of the sample has been considered in study design.	Evidence of consideration of sample size in terms of saturation/information redundancy or to fit generic analytical requirements	Explicit statement of data being gathered until information redundancy/saturation was reached or to fit exact calculations for analytical requirements.
5	Does it have Representative sample of target group of a reasonable size	3	No statement of target group	Sample is limited but represents some of the target group or representative but very small.	Sample is somewhat diverse but not entirely representative, e.g. inclusive of all age groups, experience but only one workplace. Requires discussion of target population to determine what sample is required to be representative	S ample includes individuals to represent a cross section of the target population, considering factors such as experience, age and workplace.
6	Does it have Description of procedure for data collection	3	No mention at all	Very basic and brief outline of data collection procedure,<br>e.g. 'using a questionnaire distributed to staff'.	States each stage of data collection procedure but with limited detail, or states some stages in details but omits others.	Detailed description of each stage of the data collection procedure, including when, where and how data were gathered.
7	Does it have Rationale for choice of data collection tool(s)	3	No mention at all	Very limited explanation for choice of data collection tool(s).	Basic explanation of rationale for choice of data collection tool(s), e.g. based on use in a prior similar study.	Detailed explanation of rationale for choice of data collection tool(s), e.g. relevance to the study aims and assessments of tool quality either statistically, e.g. for reliability & validity, or relevant qualitative assessment.
8	Does it have Detailed recruitment data	3	No mention at all	Minimal recruitment data, e.g. no. of questionnaire	Some recruitment information	Complete data

				sent<br>and no. returned.	but not complete account of the recruitment process, e.g. recruitment figures but no information on strategy used.	regarding no. approached, no. recruited, attrition data where relevant, method of recruitment.
9	Does it have Statistical assessment of reliability and validity of measurement tool(s)<br>(Quantitative only)	n/a	No mention at all	Reliability and validity of measurement tool(s) discussed, but not statistically assessed.	Some attempt to assess reliability and validity of measurement tool(s) but insufficient, e.g. attempt to establish test–retest reliability is unsuccessful but no action is taken.	Suitable and thorough statistical assessment of reliability and validity of measurement tool(s) with reference to the quality of evidence as a result of the measures used.
10	Does it have Fit between stated research question and method of data collection<br>(Quantitative)	n/a	No research question stated	Method of data collection can only address some aspects of the research question.	Method of data collection can address the research question but there is a more suitable alternative that could have been used or used in addition.	Method of data collection selected is the most suitable approach to attempt answer the research question
11	Does it have Fit between stated research question and format and content of data collection tool e.g. interview schedule<br>(Qualitative)	3	No research question stated	Structure and/or content only suitable to address the research question in some aspects or superficially.	Structure & content allows for data to be gathered broadly addressing the stated research question(s) but could benefit from greater detail.	Structure & content allows for detailed data to be gathered around all relevant issues required to address the stated research question(s).
12	Does it have Fit between research question and method of analysis	3	No mention at all	Method of analysis can only address the research question basically or broadly.	Method of analysis can address the research question but there is a more suitable alternative that could have been used or used in addition to offer greater detail.	Method of analysis selected is the most suitable approach to attempt answer the research question in detail, e.g. for qualitative IPA preferable for experiences vs. content analysis to elicit frequency of occurrence of events, etc.
13	Does it have Good justification for analytical method selected	3	No mention at all	Basic explanation for choice of analytical method	Fairly detailed explanation of choice of analytical method	Detailed explanation for choice of analytical method<br>based on nature of research question(s)
14	Does it have Assessment of reliability of analytical process<br>(Qualitative only)	3	No mention at all	More than one researcher involved in the analytical process but no further reliability assessment.	Limited attempt to assess reliability, e.g. reliance on one method.	Use of a range of methods to assess reliability, e.g. triangulation, multiple researchers, varying research backgrounds
15	Does it have Evidence of user involvement in design	0	No mention at all	Use of pilot study but no involvement in planning stages of study design.	Pilot study with feedback from users informing changes to the design.	Explicit consultation with steering group or statement or formal consultation with users in planning of study design.
16	Does it have Strengths and limitations critically discussed	3	No mention at all	Very limited mention of strengths and limitations with omissions of many key issues	Discussion of some of the key strengths and weaknesses of the study but not complete	Discussion of strengths and limitations of all aspects of study including design, measures, procedure, sample & analysis.
	Total score	39/42				
		93%				

Gibson et al (2020). The mental health impacts of climate change: Findings from a Pacific Island atoll nation.						
	Criteria	Rate	0 = Not at all	1 = Very slightly	2 = Moderately	3 = Complete
1	Does this study have Explicit theoretical framework	1	No mention at all	Reference to broad theoretical basis.	Reference to a specific theoretical basis.	Does this study have Explicit statement of theoretical framework and/or constructs applied to the research.
2	Does it have Statement of aims/objectives in main body of report	3	No mention at all	General reference to aim/objective at some point in the report including abstract.	Reference to broad aims/objectives in main body of report.	Explicit statement of aims/objectives in main body of report.
3	Does it have Clear description of research setting	3	No mention at all	General description of research area and background, e.g. 'in primary care'.	General description of research problem in the target population, e.g. 'among GPs in primary care'.	Specific description of the research problem and target population in the context of the study, e.g. nurses and doctors from GP practices in the east midlands.
4	Does it have Evidence of sample size considered in terms of analysis	3	No mention at all	Basic explanation for choice of sample size. Evidence that size of the sample has been considered in study design.	Evidence of consideration of sample size in terms of saturation/information redundancy or to fit generic analytical requirements	Explicit statement of data being gathered until information redundancy/saturation was reached or to fit exact calculations for analytical requirements.
5	Does it have Representative sample of target group of a reasonable size	2	No statement of target group	Sample is limited but represents some of the target group or representative but very small.	Sample is somewhat diverse but not entirely representative, e.g. inclusive of all age groups, experience but only one workplace. Requires discussion of target population to determine what sample is required to be representative	S ample includes individuals to represent a cross section of the target population, considering factors such as experience, age and workplace.
6	Does it have Description of procedure for data collection	3	No mention at all	Very basic and brief outline of data collection procedure, e.g. 'using a questionnaire distributed to staff'.	States each stage of data collection procedure but with limited detail, or states some stages in details but omits others.	Detailed description of each stage of the data collection procedure, including when, where and how data were gathered.
7	Does it have Rationale for choice of data collection tool(s)	3	No mention at all	Very limited explanation for choice of data collection tool(s).	Basic explanation of rationale for choice of data collection tool(s), e.g. based on use in a prior similar study.	Detailed explanation of rationale for choice of data collection tool(s), e.g. relevance to the study aims and assessments of tool quality either statistically, e.g. for reliability & validity, or relevant qualitative assessment.
8	Does it have Detailed recruitment data	1	No mention at all	Minimal recruitment data, e.g. no. of questionnaire sent and no. returned.	Some recruitment information but not complete account of the recruitment process, e.g. recruitment figures but no information on strategy used.	Complete data regarding no. approached, no. recruited, attrition data where relevant, method

						of recruitment.
9	Does it have Statistical assessment of reliability and validity of measurement tool(s) (Quantitative only)	3	No mention at all	Reliability and validity of measurement tool(s) discussed, but not statistically assessed.	Some attempt to assess reliability and validity of measurement tool(s) but insufficient, e.g. attempt to establish test–retest reliability is unsuccessful but no action is taken.	Suitable and thorough statistical assessment of reliability and validity of measurement tool(s) with reference to the quality of evidence as a result of the measures used.
10	Does it have Fit between stated research question and method of data collection (Quantitative)	3	No research question stated	Method of data collection can only address some aspects of the research question.	Method of data collection can address the research question but there is a more suitable alternative that could have been used or used in addition.	Method of data collection selected is the most suitable approach to attempt answer the research question
11	Does it have Fit between stated research question and format and content of data collection tool e.g. interview schedule (Qualitative	3	No research question stated	Structure and/or content only suitable to address the research question in some aspects or superficially.	Structure & content allows for data to be gathered broadly addressing the stated research question(s) but could benefit from greater detail.	Structure & content allows for detailed data to be gathered around all relevant issues required to address the stated research question(s).
12	Does it have Fit between research question and method of analysis	3	No mention at all	Method of analysis can only address the research question basically or broadly.	Method of analysis can address the research question but there is a more suitable alternative that could have been used or used in addition to offer greater detail.	Method of analysis selected is the most suitable approach to attempt answer the research question in detail, e.g. for qualitative IPA preferable for experiences vs. content analysis to elicit frequency of occurrence of events, etc.
13	Does it have Good justification for analytical method selected	3	No mention at all	Basic explanation for choice of analytical method	Fairly detailed explanation of choice of analytical method	Detailed explanation for choice of analytical method based on nature of research question(s)
14	Does it have Assessment of reliability of analytical process (Qualitative only)	1	No mention at all	More than one researcher involved in the analytical process but no further reliability assessment.	Limited attempt to assess reliability, e.g. reliance on one method.	Use of a range of methods to assess reliability, e.g. triangulation, multiple researchers, varying research backgrounds
15	Does it have Evidence of user involvement in design	3	No mention at all	Use of pilot study but no involvement in planning stages of study design.	Pilot study with feedback from users informing changes to the design.	Explicit consultation with steering group or statement or formal consultation with users in planning of study design.
16	Does it have Strengths and limitations critically discussed	3	No mention at all	Very limited mention of strengths and limitations with omissions of many key issues	Discussion of some of the key strengths and weaknesses of the study but not complete	Discussion of strengths and limitations of all aspects of study including design, measures, procedure, sample & analysis.
	Total score	41/48				
		85%				

Gibson et al (2021) Piloting a scalable, post-trauma psychosocial intervention in Tuvalu: the Skills for Life Adjustment and Resilience (SOLAR) program						
	Criteria	**Rate**	0 = Not at all	1 = Very slightly	2 = Moderately	3 = Complete
1	Does this study have Explicit theoretical framework	3	No mention at all	Reference to broad theoretical basis.	Reference to a specific theoretical basis.	Does this study have Explicit statement of theoretical framework and/or constructs applied to the research.
2	Does it have Statement of aims/objectives in main body of report	3	No mention at all	General reference to aim/objective at some point in the report including abstract.	Reference to broad aims/objectives in main body of report.	Explicit statement of aims/objectives in main body of report.
3	Does it have Clear description of research setting	3	No mention at all	General description of research area and background, e.g. 'in primary care'.	General description of research problem in the target population, e.g. 'among GPs in primary care'.	Specific description of the research problem and target population in the context of the study, e.g. nurses and doctors from GP practices in the east midlands.
4	Does it have Evidence of sample size considered in terms of analysis	3	No mention at all	Basic explanation for choice of sample size. Evidence that size of the sample has been considered in study design.	Evidence of consideration of sample size in terms of saturation/information redundancy or to fit generic analytical requirements	Explicit statement of data being gathered until information redundancy/saturation was reached or to fit exact calculations for analytical requirements.
5	Does it have Representative sample of target group of a reasonable size	3	No statement of target group	Sample is limited but represents some of the target group or representative but very small.	Sample is somewhat diverse but not entirely representative, e.g. inclusive of all age groups, experience but only one workplace. Requires discussion of target population to determine what sample is required to be representative	S ample includes individuals to represent a cross section of the target population, considering factors such as experience, age and workplace.
6	Does it have Description of procedure for data collection	1	No mention at all	Very basic and brief outline of data collection procedure, e.g. 'using a questionnaire distributed to staff'.	States each stage of data collection procedure but with limited detail, or states some stages in details but omits others.	Detailed description of each stage of the data collection procedure, including when, where and how data were gathered.
7	Does it have Rationale for choice of data collection tool(s)	0	No mention at all	Very limited explanation for choice of data collection tool(s).	Basic explanation of rationale for choice of data collection tool(s), e.g. based on use in a prior similar	Detailed explanation of rationale for choice of data collection tool(s), e.g. relevance to the study aims and assessments of tool quality either statistically, e.g. for reliability & validity, or relevant qualitative

					study.	assessment.
8	Does it have Detailed recruitment data	3	No mention at all	Minimal recruitment data, e.g. no. of questionnaire sent and no. returned.	Some recruitment information but not complete account of the recruitment process, e.g. recruitment figures but no information on strategy used.	Complete data regarding no. approached, no. recruited, attrition data where relevant, method of recruitment.
9	Does it have Statistical assessment of reliability and validity of measurement tool(s) (Quantitative only)	3	No mention at all	Reliability and validity of measurement tool(s) discussed, but not statistically assessed.	Some attempt to assess reliability and validity of measurement tool(s) but insufficient, e.g. attempt to establish test–retest reliability is unsuccessful but no action is taken.	Suitable and thorough statistical assessment of reliability and validity of measurement tool(s) with reference to the quality of evidence as a result of the measures used.
10	Does it have Fit between stated research question and method of data collection (Quantitative)	3	No research question stated	Method of data collection can only address some aspects of the research question.	Method of data collection can address the research question but there is a more suitable alternative that could have been used or used in addition.	Method of data collection selected is the most suitable approach to attempt answer the research question
11	Does it have Fit between stated research question and format and content of data collection tool e.g. interview schedule (Qualitative)	n/a	No research question stated	Structure and/or content only suitable to address the research question in some aspects or superficially.	Structure & content allows for data to be gathered broadly addressing the stated research question(s) but could benefit from greater detail.	Structure & content allows for detailed data to be gathered around all relevant issues required to address the stated research question(s).
12	Does it have Fit between research question and method of analysis	3	No mention at all	Method of analysis can only address the research question basically or broadly.	Method of analysis can address the research question but there is a more suitable alternative that could have been used or used in addition to offer greater detail.	Method of analysis selected is the most suitable approach to attempt answer the research question in detail, e.g. for qualitative IPA preferable for experiences vs. content analysis to elicit frequency of occurrence of events, etc.
13	Does it have Good justification for analytical method selected	3	No mention at all	Basic explanation for choice of analytical method	Fairly detailed explanation of choice of analytical method	Detailed explanation for choice of analytical method based on nature of research question(s)
14	Does it have Assessment of reliability	n/a	No mention	More than one researcher involved in the analytical	Limited attempt	Use of a range of methods to assess

	of analytical process (Qualitative only)		at all	process but no further reliability assessment.	to assess reliability, e.g. reliance on one method.	reliability, e.g. triangulation, multiple researchers, varying research backgrounds	
15	Does it have Evidence of user involvement in design	1	No mention at all	Use of pilot study but no involvement in planning stages of study design.	Pilot study with feedback from users informing changes to the design.	Explicit consultation with steering group or statement or formal consultation with users in planning of study design.	
16	Does it have Strengths and limitations critically discussed	3	No mention at all	Very limited mention of strengths and limitations with omissions of many key issues	Discussion of some of the key strengths and weaknesses of the study but not complete	Discussion of strengths and limitations of all aspects of study including design, measures, procedure, sample & analysis.	
	Total score	35/42					
		83%					

Furusawa et. al (2021) Health and well-being in small island communities: a cross-sectional study in the Solomon Islands						
	Criteria	**Rate**	0 = Not at all	1 = Very slightly	2 = Moderately	3 = Complete
1	Does this study have Explicit theoretical framework	0	No mention at all	Reference to broad theoretical basis.	Reference to a specific theoretical basis.	Does this study have Explicit statement of theoretical framework and/or constructs applied to the research.
2	Statement of aims/objectives in main body of report					
	3	No mention at all	General reference to aim/objective at some point in the report including abstract.	Reference to broad aims/objectives in main body of report.	Explicit statement of aims/objectives in main body of report.	
3	Clear description of research setting	3	No mention at all	General description of research area and background,		
e.g. 'in primary care'.	General description of research problem in the target population, e.g. 'among GPs in primary care'.	Specific description of the research problem and target population in the context of the study, e.g. nurses and doctors from GP practices in the east midlands.				
4	Evidence of sample size considered in terms of analysis	3	No mention at all	Basic explanation for choice of sample size. Evidence		
that size of the sample has been considered in study design.	Evidence of consideration of sample size in terms of saturation/information redundancy or to fit generic analytical requirements	Explicit statement of data being gathered until information redundancy/saturation was reached or to fit exact calculations for analytical requirements.				
5	Representative sample of target group of a reasonable size	2	No statement of target group	Sample is limited but represents some of the target group or representative but very small.		
	Sample is somewhat diverse but not entirely representative, e.g. inclusive of all age groups, experience but only one workplace. Requires discussion of target population to determine what sample is required to be representative	Sample includes individuals to represent a cross section of the target population, considering factors such as experience, age and workplace.				
6	Description of procedure for data collection	3	No mention at all	Very basic and brief outline of data		

				collection procedure,		
e.g. 'using a questionnaire distributed to staff'.	States each stage of data collection procedure but with limited detail, or states some stages in details but omits others.	Detailed description of each stage of the data collection procedure, including when, where and how data were gathered.				
7	Rationale for choice of data collection tool(s)	3	No mention at all	Very limited explanation for choice of data collection		
tool(s).	Basic explanation of rationale for choice of data collection tool(s), e.g. based on use in a prior similar study.					
	Detailed explanation of rationale for choice of data collection tool(s), e.g. relevance to the study aims and assessments of tool quality either statistically, e.g. for reliability & validity, or relevant qualitative assessment.					
8	Detailed recruitment data	3	No mention at all	Minimal recruitment data, e.g. no. of questionnaire sent		
and no. returned.	Some recruitment information but not complete account of the recruitment process, e.g. recruitment figures but no information on strategy used.	Complete data regarding no. approached, no. recruited, attrition data where relevant, method of recruitment.				
9	Statistical assessment of reliability and validity of measurement tool(s)					
(Quantitative only)	3	No mention at all	Reliability and validity of measurement tool(s) discussed, but not statistically assessed.	Some attempt to assess reliability and validity of measurement tool(s) but insufficient, e.g. attempt to establish test–retest reliability is unsuccessful but no action is taken.	Suitable and thorough statistical assessment of reliability and validity of measurement tool(s) with reference to the quality of evidence as a result of the measures used.	
10	Fit between stated research question and method of data collection					
(Quantitative)	3	No research question stated	Method of data collection can only address some aspects of the research question.	Method of data collection can address the research question but there is a more suitable alternative that could have been used or used in addition.	Method of data collection selected is the most suitable approach to attempt answer the research question	
11	Fit between stated research question and format and content of data collection tool e.g. interview schedule					
(Qualitative	n/a	No research question stated	Structure and/or content only suitable to address the research question in some aspects or superficially.			
	Structure & content allows for data to be gathered broadly addressing the stated research question(s) but could benefit from greater detail.					
	Structure & content allows for detailed data to be gathered around all relevant issues required to address the stated research question(s).					
12	Fit between research question and method of analysis	2	No mention at all	Method of analysis can only address the research		
question basically or broadly.	Method of analysis can address the research question but there is a more suitable alternative that could have been used or used in addition to offer greater detail.					
	Method of analysis selected is the most suitable approach to attempt answer the research question in detail, e.g. for qualitative IPA preferable for experiences vs. content analysis to elicit frequency of occurrence of events, etc.					
13	Good justification for analytical method selected	3	No mention at all	Basic explanation for choice of analytical method	Fairly detailed explanation of choice of analytical method	Detailed explanation for choice of analytical method
based on nature of research question(s)						
14	Assessment of reliability of analytical process					

(Qualitative only)	n/a	No mention at all	More than one researcher involved in the analytical			
process but no further reliability assessment.	Limited attempt to assess reliability, e.g. reliance on one method.	Use of a range of methods to assess reliability, e.g. triangulation, multiple researchers, varying research backgrounds				
15	Evidence of user involvement in design	3	No mention at all	Use of pilot study but no involvement in planning stages of study design.	Pilot study with feedback from users informing changes to the design.	Explicit consultation with steering group or statement or formal consultation with users in planning of study design.
16	Strengths and limitations critically discussed	3	No mention at all	Very limited mention of strengths and limitations with omissions of many key issues	Discussion of some of the key strengths and weaknesses of the study but not complete	Discussion of strengths and limitations of all aspects of study including design, measures, procedure, sample & analysis.
	Total score	37/42				
		88%				

Clissold et. al (2023) Experiencing and responding to extreme weather: lessons from the Cook Islands.						
	Criteria	**Rate**	0 = Not at all	1 = Very slightly	2 = Moderately	3 = Complete
1	Does this study have Explicit theoretical framework	0	No mention at all	Reference to broad theoretical basis.	Reference to a specific theoretical basis.	Does this study have Explicit statement of theoretical framework and/or constructs applied to the research.
2	Does it have Statement of aims/objectives in main body of report					
	0	No mention at all	General reference to aim/objective at some point in the report including abstract.	Reference to broad aims/objectives in main body of report.	Explicit statement of aims/objectives in main body of report.	
3	Does it have Clear description of research setting	3	No mention at all	General description of research area and background,		
e.g. ‘in primary care’.	General description of research problem in the target population, e.g. ‘among GPs in primary care’.	Specific description of the research problem and target population in the context of the study, e.g. nurses and doctors from GP practices in the east midlands.				
4	Does it have Evidence of sample size considered in terms of analysis	1	No mention at all	Basic explanation for choice of sample size. Evidence		
that size of the sample has been considered in study design.	Evidence of consideration of sample size in terms of saturation/information redundancy or to fit generic analytical requirements	Explicit statement of data being gathered until information redundancy/saturation was reached or to fit exact calculations for analytical requirements.				
5	Does it have Representative sample of target group of a reasonable size	1	No statement of target group	Sample is limited but represents some of the target group or representative but very small.		
	Sample is somewhat diverse but not entirely representative, e.g. inclusive of all age groups, experience but only one workplace. Requires	S ample includes individuals to represent a cross section of the target population, considering factors such as experience, age				

	discussion of target population to determine what sample is required to be representative	and workplace.				
6	Does it have Description of procedure for data collection	3	No mention at all	Very basic and brief outline of data collection procedure,		
e.g. 'using a questionnaire distributed to staff'.	States each stage of data collection procedure but with limited detail, or states some stages in details but omits others.	Detailed description of each stage of the data collection procedure, including when, where and how data were gathered.				
7	Does it have Rationale for choice of data collection tool(s)	3	No mention at all	Very limited explanation for choice of data collection		
tool(s).	Basic explanation of rationale for choice of data collection tool(s), e.g. based on use in a prior similar study.					
	Detailed explanation of rationale for choice of data collection tool(s), e.g. relevance to the study aims and assessments of tool quality either statistically, e.g. for reliability & validity, or relevant qualitative assessment.					
8	Does it have Detailed recruitment data	0	No mention at all	Minimal recruitment data, e.g. no. of questionnaire sent		
and no. returned.	Some recruitment information but not complete account of the recruitment process, e.g. recruitment figures but no information on strategy used.	Complete data regarding no. approached, no. recruited, attrition data where relevant, method of recruitment.				
9	Does it have Statistical assessment of reliability and validity of measurement tool(s)					
(Quantitative only)	n/a	No mention at all	Reliability and validity of measurement tool(s) discussed, but not statistically assessed.	Some attempt to assess reliability and validity of measurement tool(s) but insufficient, e.g. attempt to establish test–retest reliability is unsuccessful but no action is taken.	Suitable and thorough statistical assessment of reliability and validity of measurement tool(s) with reference to the quality of evidence as a result of the measures used.	
10	Does it have Fit between stated research question and method of data collection					
(Quantitative)	n/a	No research question stated	Method of data collection can only address some aspects of the research question.	Method of data collection can address the research question but there is a more suitable alternative that could have been used or used in addition.	Method of data collection selected is the most suitable approach to attempt answer the research question	
11	Does it have Fit between stated research question and format and content of data collection tool e.g. interview schedule					
(Qualitative	3	No research question stated	Structure and/or content only suitable to address the research question in some aspects or superficially.			
	Structure & content allows for data to be gathered broadly addressing the stated research question(s) but could benefit from greater detail.					
	Structure & content allows for detailed data to be gathered around all relevant issues required to address the stated research question(s).					
12	Does it have Fit between research question and method of analysis	3	No mention at all	Method of analysis can only address the research		
question basically or broadly.	Method of analysis can address the research question but there is a more suitable alternative that could have been used or used in addition to offer greater detail.					
	Method of analysis selected is the most suitable approach to attempt answer the research question in detail, e.g. for qualitative IPA preferable for experiences vs. content analysis to elicit frequency of occurrence of events, etc.					
13	Does it have Good justification for analytical method selected	3	No mention at all	Basic explanation for choice of analytical method	Fairly detailed explanation of choice of analytical method	Detailed explanation for choice of analytical method

based on nature of research question(s)						
14	Does it have Assessment of reliability of analytical process					
(Qualitative only)	0	No mention at all	More than one researcher involved in the analytical			
process but no further reliability assessment.	Limited attempt to assess reliability, e.g. reliance on one method.	Use of a range of methods to assess reliability, e.g. triangulation, multiple researchers, varying research backgrounds				
15	Does it have Evidence of user involvement in design	3	No mention at all	Use of pilot study but no involvement in planning stages of study design.	Pilot study with feedback from users informing changes to the design.	Explicit consultation with steering group or statement or formal consultation with users in planning of study design.
16	Does it have Strengths and limitations critically discussed	3	No mention at all	Very limited mention of strengths and limitations with omissions of many key issues	Discussion of some of the key strengths and weaknesses of the study but not complete	Discussion of strengths and limitations of all aspects of study including design, measures, procedure, sample & analysis.
	Total score	26/42				
		62%				

Supplementary File 2 : Results of the QATSDD analysis

Asugeni et al (2015) Mental health issues from rising sea level in a remote coastal region of the Solomon Islands: Current and future.						
	Criteria	**Rate**	0 = Not at all	1 = Very slightly	2 = Moderately	3 = Complete
1	Does this study have Explicit theoretical framework	0	No mention at all	Reference to broad theoretical basis.	Reference to a specific theoretical basis.	Does this study have Explicit statement of theoretical framework and/or constructs applied to the research.
2	Does it have Statement of aims/objectives in main body of report	3	No mention at all	General reference to aim/objective at some point in the report including abstract.	Reference to broad aims/objectives in main body of report.	Explicit statement of aims/objectives in main body of report.
3	Does it have Clear description of research setting	1	No mention at all	General description of research area and background, e.g. 'in primary care'.	General description of research problem in the target population, e.g. 'among GPs in primary care'.	Specific description of the research problem and target population in the context of the study, e.g. nurses and doctors from GP practices in the east midlands.
4	Does it have Evidence of sample size considered in terms of analysis	1	No mention at all	Basic explanation for choice of sample size. Evidence that size of the sample has been considered in study design.	Evidence of consideration of sample size in terms of saturation/information redundancy or to fit generic analytical requirements	Explicit statement of data being gathered until information redundancy/saturation was reached or to fit exact calculations for analytical requirements.
5	Does it have Representative sample of target group of a reasonable size	2	No statement of target group	Sample is limited but represents some of the target group or representative but very small.	Sample is somewhat diverse but not entirely representative, e.g. inclusive of all age groups, experience but only one workplace. Requires discussion of target population to determine what sample is required to be representative	S ample includes individuals to represent a cross section of the target population, considering factors such as experience, age and workplace.
6	Does it have Description of procedure for data collection	3	No mention at all	Very basic and brief outline of data collection procedure,<br>e.g. 'using a questionnaire distributed to staff'.	States each stage of data collection procedure but with limited detail, or states some stages in details but omits others.	Detailed description of each stage of the data collection procedure, including when, where and how data were gathered.
7	Does it have Rationale for choice of data collection tool(s)	2	No mention at all	Very limited explanation for choice of data collection tool(s).	Basic explanation of rationale for choice of data collection tool(s), e.g. based on use in a prior similar study.	Detailed explanation of rationale for choice of data collection tool(s), e.g. relevance to the study aims and assessments of tool quality either statistically, e.g. for reliability & validity, or relevant qualitative assessment.
8	Does it have Detailed recruitment data	3	No mention at all	Minimal recruitment data, e.g. no. of questionnaire sent<br>and no. returned.	Some recruitment information but not complete account of the recruitment process, e.g. recruitment figures but no information on strategy used.	Complete data regarding no. approached, no. recruited, attrition data where relevant, method of recruitment.
9	Does it have Statistical assessment of reliability and validity of measurement tool(s)<br>(Quantitative only)	0	No mention at all	Reliability and validity of measurement tool(s) discussed, but not statistically assessed.	Some attempt to assess reliability and validity of measurement tool(s) but insufficient, e.g. attempt to establish test–retest reliability is unsuccessful but no action	Suitable and thorough statistical assessment of reliability and validity of measurement tool(s) with reference to the quality of evidence as a result of the measures used.

					is taken.	
10	Does it have Fit between stated research question and method of data collection (Quantitative)	3	No research question stated	Method of data collection can only address some aspects of the research question.	Method of data collection can address the research question but there is a more suitable alternative that could have been used or used in addition.	Method of data collection selected is the most suitable approach to attempt answer the research question
11	Does it have Fit between stated research question and format and content of data collection tool e.g. interview schedule (Qualitative)	3	No research question stated	Structure and/or content only suitable to address the research question in some aspects or superficially.	Structure & content allows for data to be gathered broadly addressing the stated research question(s) but could benefit from greater detail.	Structure & content allows for detailed data to be gathered around all relevant issues required to address the stated research question(s).
12	Does it have Fit between research question and method of analysis	2	No mention at all	Method of analysis can only address the research question basically or broadly.	Method of analysis can address the research question but there is a more suitable alternative that could have been used or used in addition to offer greater detail.	Method of analysis selected is the most suitable approach to attempt answer the research question in detail, e.g. for qualitative IPA preferable for experiences vs. content analysis to elicit frequency of occurrence of events, etc.
13	Does it have Good justification for analytical method selected	3	No mention at all	Basic explanation for choice of analytical method	Fairly detailed explanation of choice of analytical method	Detailed explanation for choice of analytical method based on nature of research question(s)
14	Does it have Assessment of reliability of analytical process (Qualitative only)	2	No mention at all	More than one researcher involved in the analytical process but no further reliability assessment.	Limited attempt to assess reliability, e.g. reliance on one method.	Use of a range of methods to assess reliability, e.g. triangulation, multiple researchers, varying research backgrounds
15	Does it have Evidence of user involvement in design	3	No mention at all	Use of pilot study but no involvement in planning stages of study design.	Pilot study with feedback from users informing changes to the design.	Explicit consultation with steering group or statement or formal consultation with users in planning of study design.
16	Does it have Strengths and limitations critically discussed	3	No mention at all	Very limited mention of strengths and limitations with omissions of many key issues	Discussion of some of the key strengths and weaknesses of the study but not complete	Discussion of strengths and limitations of all aspects of study including design, measures, procedure, sample & analysis.
	Total score	34/48				
		71%				

Gibson et al (2019). Distressing encounters in the context of climate change: Idioms of distress, determinants, and responses to distress in Tuvalu						
	Criteria	**Rate**	0 = Not at all	1 = Very slightly	2 = Moderately	3 = Complete
1	Does this study have Explicit theoretical framework	3	No mention at all	Reference to broad theoretical basis.	Reference to a specific theoretical basis.	Does this study have Explicit statement of theoretical framework and/or constructs applied to the research.
2	Does it have Statement of aims/objectives in main body of report	3	No mention at all	General reference to aim/objective at some point in the report including abstract.	Reference to broad aims/objectives in main body of report.	Explicit statement of aims/objectives in main body of report.
3	Does it have Clear description of research setting	3	No mention at all	General description of research area and background, e.g. 'in primary care'.	General description of research problem in the target population, e.g. 'among GPs in primary care'.	Specific description of the research problem and target population in the context of the study, e.g. nurses and doctors from GP practices in the east midlands.
4	Does it have Evidence of sample size considered in terms of analysis	3	No mention at all	Basic explanation for choice of sample size. Evidence that size of the sample has been considered in study design.	Evidence of consideration of sample size in terms of saturation/information redundancy or to fit generic analytical requirements	Explicit statement of data being gathered until information redundancy/saturation was reached or to fit exact calculations for analytical requirements.
5	Does it have Representative sample of target group of a reasonable size	3	No statement of target group	Sample is limited but represents some of the target group or representative but very small.	Sample is somewhat diverse but not entirely representative, e.g. inclusive of all age groups, experience but only one workplace. Requires discussion of target population to determine what sample is required to be representative	S ample includes individuals to represent a cross section of the target population, considering factors such as experience, age and workplace.
6	Does it have Description of procedure for data collection	3	No mention at all	Very basic and brief outline of data collection procedure, e.g. 'using a questionnaire distributed to staff'.	States each stage of data collection procedure but with limited detail, or states some stages in details but omits others.	Detailed description of each stage of the data collection procedure, including when, where and how data were gathered.
7	Does it have Rationale for choice of data collection tool(s)	3	No mention at all	Very limited explanation for choice of data collection tool(s).	Basic explanation of rationale for choice of data collection tool(s), e.g. based on use in a prior similar study.	Detailed explanation of rationale for choice of data collection tool(s), e.g. relevance to the study aims and assessments of tool quality either statistically, e.g. for reliability & validity, or relevant qualitative assessment.
8	Does it have Detailed recruitment data	3	No mention at all	Minimal recruitment data, e.g. no. of questionnaire sent and no. returned.	Some recruitment information but not complete account of the recruitment process, e.g. recruitment figures but no information on strategy used.	Complete data regarding no. approached, no. recruited, attrition data where relevant, method of recruitment.
9	Does it have Statistical assessment of reliability and validity of measurement tool(s) (Quantitative only)	n/a	No mention at all	Reliability and validity of measurement tool(s) discussed, but not statistically assessed.	Some attempt to assess reliability and validity of measurement tool(s) but insufficient, e.g. attempt to establish test–retest reliability is unsuccessful but no action is taken.	Suitable and thorough statistical assessment of reliability and validity of measurement tool(s) with reference to the quality of evidence as a result of the measures used.

10	Does it have Fit between stated research question and method of data collection (Quantitative)	n/a	No research question stated	Method of data collection can only address some aspects of the research question.	Method of data collection can address the research question but there is a more suitable alternative that could have been used or used in addition.	Method of data collection selected is the most suitable approach to attempt answer the research question
11	Does it have Fit between stated research question and format and content of data collection tool e.g. interview schedule (Qualitative)	3	No research question stated	Structure and/or content only suitable to address the research question in some aspects or superficially.	Structure & content allows for data to be gathered broadly addressing the stated research question(s) but could benefit from greater detail.	Structure & content allows for detailed data to be gathered around all relevant issues required to address the stated research question(s).
12	Does it have Fit between research question and method of analysis	3	No mention at all	Method of analysis can only address the research question basically or broadly.	Method of analysis can address the research question but there is a more suitable alternative that could have been used or used in addition to offer greater detail.	Method of analysis selected is the most suitable approach to attempt answer the research question in detail, e.g. for qualitative IPA preferable for experiences vs. content analysis to elicit frequency of occurrence of events, etc.
13	Does it have Good justification for analytical method selected	3	No mention at all	Basic explanation for choice of analytical method	Fairly detailed explanation of choice of analytical method	Detailed explanation for choice of analytical method based on nature of research question(s)
14	Does it have Assessment of reliability of analytical process (Qualitative only)	3	No mention at all	More than one researcher involved in the analytical process but no further reliability assessment.	Limited attempt to assess reliability, e.g. reliance on one method.	Use of a range of methods to assess reliability, e.g. triangulation, multiple researchers, varying research backgrounds
15	Does it have Evidence of user involvement in design	0	No mention at all	Use of pilot study but no involvement in planning stages of study design.	Pilot study with feedback from users informing changes to the design.	Explicit consultation with steering group or statement or formal consultation with users in planning of study design.
16	Does it have Strengths and limitations critically discussed	3	No mention at all	Very limited mention of strengths and limitations with omissions of many key issues	Discussion of some of the key strengths and weaknesses of the study but not complete	Discussion of strengths and limitations of all aspects of study including design, measures, procedure, sample & analysis.
	Total score	39/42				
		93%				

Gibson et al (2020). The mental health impacts of climate change: Findings from a Pacific Island atoll nation.						
	Criteria	**Rate**	0 = Not at all	1 = Very slightly	2 = Moderately	3 = Complete
1	Does this study have Explicit theoretical framework	1	No mention at all	Reference to broad theoretical basis.	Reference to a specific theoretical basis.	Does this study have Explicit statement of theoretical framework and/or constructs applied to the research.
2	Does it have Statement of aims/objectives in main body of report	3	No mention at all	General reference to aim/objective at some point in the report including abstract.	Reference to broad aims/objectives in main body of report.	Explicit statement of aims/objectives in main body of report.
3	Does it have Clear description of research setting	3	No mention at all	General description of research area and background, e.g. 'in primary care'.	General description of research problem in the target population, e.g. 'among GPs in primary care'.	Specific description of the research problem and target population in the context of the study, e.g. nurses and doctors from GP practices in the east midlands.
4	Does it have Evidence of sample size considered in terms of analysis	3	No mention at all	Basic explanation for choice of sample size. Evidence that size of the sample has been considered in study design.	Evidence of consideration of sample size in terms of saturation/information redundancy or to fit generic analytical requirements	Explicit statement of data being gathered until information redundancy/saturation was reached or to fit exact calculations for analytical requirements.
5	Does it have Representative sample of target group of a reasonable size	2	No statement of target group	Sample is limited but represents some of the target group or representative but very small.	Sample is somewhat diverse but not entirely representative, e.g. inclusive of all age groups, experience but only one workplace. Requires discussion of target population to determine what sample is required to be representative	S ample includes individuals to represent a cross section of the target population, considering factors such as experience, age and workplace.
6	Does it have Description of procedure for data collection	3	No mention at all	Very basic and brief outline of data collection procedure, e.g. 'using a questionnaire distributed to staff'.	States each stage of data collection procedure but with limited detail, or states some stages in details but omits others.	Detailed description of each stage of the data collection procedure, including when, where and how data were gathered.
7	Does it have Rationale for choice of data collection tool(s)	3	No mention at all	Very limited explanation for choice of data collection tool(s).	Basic explanation of rationale for choice of data collection tool(s), e.g. based on use in a prior similar study.	Detailed explanation of rationale for choice of data collection tool(s), e.g. relevance to the study aims and assessments of tool quality either statistically, e.g. for reliability & validity, or relevant qualitative assessment.
8	Does it have Detailed recruitment data	1	No mention at all	Minimal recruitment data, e.g. no. of questionnaire sent and no. returned.	Some recruitment information but not complete account of the recruitment process, e.g. recruitment figures but no information on strategy used.	Complete data regarding no. approached, no. recruited, attrition data where relevant, method of recruitment.
9	Does it have Statistical assessment of reliability and validity of measurement tool(s) (Quantitative only)	3	No mention at all	Reliability and validity of measurement tool(s) discussed, but not statistically assessed.	Some attempt to assess reliability and validity of measurement tool(s) but insufficient, e.g. attempt to establish test–retest reliability is unsuccessful but no action is taken.	Suitable and thorough statistical assessment of reliability and validity of measurement tool(s) with reference to the quality of evidence as a result of the measures used.
10	Does it have Fit between stated research question and	3	No research question	Method of data collection can only address some	Method of data collection can address the research	Method of data collection selected is the most suitable approach to

	method of data collection (Quantitative)		stated	aspects of the research question.	question but there is a more suitable alternative that could have been used or used in addition.	attempt answer the research question
11	Does it have Fit between stated research question and format and content of data collection tool e.g. interview schedule (Qualitative	3	No research question stated	Structure and/or content only suitable to address the research question in some aspects or superficially.	Structure & content allows for data to be gathered broadly addressing the stated research question(s) but could benefit from greater detail.	Structure & content allows for detailed data to be gathered around all relevant issues required to address the stated research question(s).
12	Does it have Fit between research question and method of analysis	3	No mention at all	Method of analysis can only address the research question basically or broadly.	Method of analysis can address the research question but there is a more suitable alternative that could have been used or used in addition to offer greater detail.	Method of analysis selected is the most suitable approach to attempt answer the research question in detail, e.g. for qualitative IPA preferable for experiences vs. content analysis to elicit frequency of occurrence of events, etc.
13	Does it have Good justification for analytical method selected	3	No mention at all	Basic explanation for choice of analytical method	Fairly detailed explanation of choice of analytical method	Detailed explanation for choice of analytical method based on nature of research question(s)
14	Does it have Assessment of reliability of analytical process (Qualitative only)	1	No mention at all	More than one researcher involved in the analytical process but no further reliability assessment.	Limited attempt to assess reliability, e.g. reliance on one method.	Use of a range of methods to assess reliability, e.g. triangulation, multiple researchers, varying research backgrounds
15	Does it have Evidence of user involvement in design	3	No mention at all	Use of pilot study but no involvement in planning stages of study design.	Pilot study with feedback from users informing changes to the design.	Explicit consultation with steering group or statement or formal consultation with users in planning of study design.
16	Does it have Strengths and limitations critically discussed	3	No mention at all	Very limited mention of strengths and limitations with omissions of many key issues	Discussion of some of the key strengths and weaknesses of the study but not complete	Discussion of strengths and limitations of all aspects of study including design, measures, procedure, sample & analysis.
	Total score	41/48				
		85%				

Gibson et al (2021) Piloting a scalable, post-trauma psychosocial intervention in Tuvalu: the Skills for Life Adjustment and Resilience (SOLAR) program						
	Criteria	**Rate**	0 = Not at all	1 = Very slightly	2 = Moderately	3 = Complete
1	Does this study have Explicit theoretical framework	3	No mention at all	Reference to broad theoretical basis.	Reference to a specific theoretical basis.	Does this study have Explicit statement of theoretical framework and/or constructs applied to the research.
2	Does it have Statement of aims/objectives in main body of report	3	No mention at all	General reference to aim/objective at some point in the report including abstract.	Reference to broad aims/objectives in main body of report.	Explicit statement of aims/objectives in main body of report.
3	Does it have Clear description of research setting	3	No mention at all	General description of research area and background, e.g. 'in primary care'.	General description of research problem in the target population, e.g. 'among GPs in primary care'.	Specific description of the research problem and target population in the context of the study, e.g. nurses and doctors from GP practices in the east midlands.
4	Does it have Evidence of sample size considered in terms of analysis	3	No mention at all	Basic explanation for choice of sample size. Evidence that size of the sample has been considered in study design.	Evidence of consideration of sample size in terms of saturation/information redundancy or to fit generic analytical requirements	Explicit statement of data being gathered until information redundancy/saturation was reached or to fit exact calculations for analytical requirements.
5	Does it have Representative sample of target group of a reasonable size	3	No statement of target group	Sample is limited but represents some of the target group or representative but very small.	Sample is somewhat diverse but not entirely representative, e.g. inclusive of all age groups, experience but only one workplace. Requires discussion of target population to determine what sample is required to be representative	S ample includes individuals to represent a cross section of the target population, considering factors such as experience, age and workplace.
6	Does it have Description of procedure for data collection	1	No mention at all	Very basic and brief outline of data collection procedure, e.g. 'using a questionnaire distributed to staff'.	States each stage of data collection procedure but with limited detail, or states some stages in details but omits others.	Detailed description of each stage of the data collection procedure, including when, where and how data were gathered.
7	Does it have Rationale for choice of data collection tool(s)	0	No mention at all	Very limited explanation for choice of data collection tool(s).	Basic explanation of rationale for choice of data collection tool(s), e.g. based on use in a prior similar study.	Detailed explanation of rationale for choice of data collection tool(s), e.g. relevance to the study aims and assessments of tool quality either statistically, e.g. for reliability & validity, or relevant qualitative assessment.
8	Does it have Detailed recruitment data	3	No mention at all	Minimal recruitment data, e.g. no. of questionnaire sent and no. returned.	Some recruitment information but not complete account of the recruitment process, e.g. recruitment figures but no information on strategy used.	Complete data regarding no. approached, no. recruited, attrition data where relevant, method of recruitment.
9	Does it have Statistical assessment of reliability and validity of measurement tool(s) (Quantitative only)	3	No mention at all	Reliability and validity of measurement tool(s) discussed, but not statistically assessed.	Some attempt to assess reliability and validity of measurement tool(s) but insufficient, e.g. attempt to establish test–retest reliability is unsuccessful but no action is taken.	Suitable and thorough statistical assessment of reliability and validity of measurement tool(s) with reference to the quality of evidence as a result of the measures used.
10	Does it have Fit between stated research question and method of data collection	3	No research question stated	Method of data collection can only address some aspects of the research question.	Method of data collection can address the research question but there is a more suitable alternative that could	Method of data collection selected is the most suitable approach to attempt answer the research question

	(Quantitative)				have been used or used in addition.	
11	Does it have Fit between stated research question and format and content of data collection tool e.g. interview schedule (Qualitative)	n/a	No research question stated	Structure and/or content only suitable to address the research question in some aspects or superficially.	Structure & content allows for data to be gathered broadly addressing the stated research question(s) but could benefit from greater detail.	Structure & content allows for detailed data to be gathered around all relevant issues required to address the stated research question(s).
12	Does it have Fit between research question and method of analysis	3	No mention at all	Method of analysis can only address the research question basically or broadly.	Method of analysis can address the research question but there is a more suitable alternative that could have been used or used in addition to offer greater detail.	Method of analysis selected is the most suitable approach to attempt answer the research question in detail, e.g. for qualitative IPA preferable for experiences vs. content analysis to elicit frequency of occurrence of events, etc.
13	Does it have Good justification for analytical method selected	3	No mention at all	Basic explanation for choice of analytical method	Fairly detailed explanation of choice of analytical method	Detailed explanation for choice of analytical method based on nature of research question(s)
14	Does it have Assessment of reliability of analytical process (Qualitative only)	n/a	No mention at all	More than one researcher involved in the analytical process but no further reliability assessment.	Limited attempt to assess reliability, e.g. reliance on one method.	Use of a range of methods to assess reliability, e.g. triangulation, multiple researchers, varying research backgrounds
15	Does it have Evidence of user involvement in design	1	No mention at all	Use of pilot study but no involvement in planning stages of study design.	Pilot study with feedback from users informing changes to the design.	Explicit consultation with steering group or statement or formal consultation with users in planning of study design.
16	Does it have Strengths and limitations critically discussed	3	No mention at all	Very limited mention of strengths and limitations with omissions of many key issues	Discussion of some of the key strengths and weaknesses of the study but not complete	Discussion of strengths and limitations of all aspects of study including design, measures, procedure, sample & analysis.
	Total score	35/42				
		83%				

Furusawa et. al (2021) Health and well-being in small island communities: a cross-sectional study in the Solomon Islands						
	Criteria	**Rate**	0 = Not at all	1 = Very slightly	2 = Moderately	3 = Complete
1	Does this study have Explicit theoretical framework	0	No mention at all	Reference to broad theoretical basis.	Reference to a specific theoretical basis.	Does this study have Explicit statement of theoretical framework and/or constructs applied to the research.
2	Statement of aims/objectives in main body of report	3	No mention at all	General reference to aim/objective at some point in the report including abstract.	Reference to broad aims/objectives in main body of report.	Explicit statement of aims/objectives in main body of report.
3	Clear description of research setting	3	No mention at all	General description of research area and background, e.g. 'in primary care'.	General description of research problem in the target population, e.g. 'among GPs in primary care'.	Specific description of the research problem and target population in the context of the study, e.g. nurses and doctors from GP practices in the east midlands.
4	Evidence of sample size considered in terms of analysis	3	No mention at all	Basic explanation for choice of sample size. Evidence that size of the sample has been considered in study design.	Evidence of consideration of sample size in terms of saturation/information redundancy or to fit generic analytical requirements	Explicit statement of data being gathered until information redundancy/saturation was reached or to fit exact calculations for analytical requirements.
5	Representative sample of target group of a reasonable size	2	No statement of target group	Sample is limited but represents some of the target group or representative but very small.	Sample is somewhat diverse but not entirely representative, e.g. inclusive of all age groups, experience but only one workplace. Requires discussion of target population to determine what sample is required to be representative	Sample includes individuals to represent a cross section of the target population, considering factors such as experience, age and workplace.
6	Description of procedure for data collection	3	No mention at all	Very basic and brief outline of data collection procedure, e.g. 'using a questionnaire distributed to staff'.	States each stage of data collection procedure but with limited detail, or states some stages in details but omits others.	Detailed description of each stage of the data collection procedure, including when, where and how data were gathered.
7	Rationale for choice of data collection tool(s)	3	No mention at all	Very limited explanation for choice of data collection tool(s).	Basic explanation of rationale for choice of data collection tool(s), e.g. based on use in a prior similar study.	Detailed explanation of rationale for choice of data collection tool(s), e.g. relevance to the study aims and assessments of tool quality either statistically, e.g. for reliability & validity, or relevant qualitative assessment.
8	Detailed recruitment data	3	No mention at all	Minimal recruitment data, e.g. no. of questionnaire sent and no. returned.	Some recruitment information but not complete account of the recruitment process, e.g. recruitment figures but no information on strategy used.	Complete data regarding no. approached, no. recruited, attrition data where relevant, method of recruitment.
9	Statistical assessment of reliability and validity of measurement tool(s) (Quantitative only)	3	No mention at all	Reliability and validity of measurement tool(s) discussed, but not statistically assessed.	Some attempt to assess reliability and validity of measurement tool(s) but insufficient, e.g. attempt to establish test–retest reliability is unsuccessful but no action is taken.	Suitable and thorough statistical assessment of reliability and validity of measurement tool(s) with reference to the quality of evidence as a result of the measures used.
10	Fit between stated research question and method of data collection (Quantitative)	3	No research question stated	Method of data collection can only address some aspects of the research question.	Method of data collection can address the research question but there is a more suitable alternative that could have been used or used in addition.	Method of data collection selected is the most suitable approach to attempt answer the research question
11	Fit between stated research question and format and	n/a	No research question	Structure and/or content only suitable to address the	Structure & content allows for data to be gathered broadly	Structure & content allows for detailed data to be gathered around all

	content of data collection tool e.g. interview schedule (Qualitative		stated	research question in some aspects or superficially.	addressing the stated research question(s) but could benefit from greater detail.	relevant issues required to address the stated research question(s).
12	Fit between research question and method of analysis	2	No mention at all	Method of analysis can only address the research question basically or broadly.	Method of analysis can address the research question but there is a more suitable alternative that could have been used or used in addition to offer greater detail.	Method of analysis selected is the most suitable approach to attempt answer the research question in detail, e.g. for qualitative IPA preferable for experiences vs. content analysis to elicit frequency of occurrence of events, etc.
13	Good justification for analytical method selected	3	No mention at all	Basic explanation for choice of analytical method	Fairly detailed explanation of choice of analytical method	Detailed explanation for choice of analytical method based on nature of research question(s)
14	Assessment of reliability of analytical process (Qualitative only)	n/a	No mention at all	More than one researcher involved in the analytical process but no further reliability assessment.	Limited attempt to assess reliability, e.g. reliance on one method.	Use of a range of methods to assess reliability, e.g. triangulation, multiple researchers, varying research backgrounds
15	Evidence of user involvement in design	3	No mention at all	Use of pilot study but no involvement in planning stages of study design.	Pilot study with feedback from users informing changes to the design.	Explicit consultation with steering group or statement or formal consultation with users in planning of study design.
16	Strengths and limitations critically discussed	3	No mention at all	Very limited mention of strengths and limitations with omissions of many key issues	Discussion of some of the key strengths and weaknesses of the study but not complete	Discussion of strengths and limitations of all aspects of study including design, measures, procedure, sample & analysis.
	Total score	37/42				
		88%				

Clissold et. al (2023) Experiencing and responding to extreme weather: lessons from the Cook Islands.						
	Criteria	**Rate**	0 = Not at all	1 = Very slightly	2 = Moderately	3 = Complete
1	Does this study have Explicit theoretical framework	0	No mention at all	Reference to broad theoretical basis.	Reference to a specific theoretical basis.	Does this study have Explicit statement of theoretical framework and/or constructs applied to the research.
2	Does it have Statement of aims/objectives in main body of report	0	No mention at all	General reference to aim/objective at some point in the report including abstract.	Reference to broad aims/objectives in main body of report.	Explicit statement of aims/objectives in main body of report.
3	Does it have Clear description of research setting	3	No mention at all	General description of research area and background, e.g. 'in primary care'.	General description of research problem in the target population, e.g. 'among GPs in primary care'.	Specific description of the research problem and target population in the context of the study, e.g. nurses and doctors from GP practices in the east midlands.
4	Does it have Evidence of sample size considered in terms of analysis	1	No mention at all	Basic explanation for choice of sample size. Evidence that size of the sample has been considered in study design.	Evidence of consideration of sample size in terms of saturation/information redundancy or to fit generic analytical requirements	Explicit statement of data being gathered until information redundancy/saturation was reached or to fit exact calculations for analytical requirements.
5	Does it have Representative sample of target group of a reasonable size	1	No statement of target group	Sample is limited but represents some of the target group or representative but very small.	Sample is somewhat diverse but not entirely representative, e.g. inclusive of all age groups, experience but only one workplace. Requires discussion of target population to determine what sample is required to be representative	S ample includes individuals to represent a cross section of the target population, considering factors such as experience, age and workplace.
6	Does it have Description of procedure for data collection	3	No mention at all	Very basic and brief outline of data collection procedure, e.g. 'using a questionnaire distributed to staff'.	States each stage of data collection procedure but with limited detail, or states some stages in details but omits others.	Detailed description of each stage of the data collection procedure, including when, where and how data were gathered.
7	Does it have Rationale for choice of data collection tool(s)	3	No mention at all	Very limited explanation for choice of data collection tool(s).	Basic explanation of rationale for choice of data collection tool(s), e.g. based on use in a prior similar study.	Detailed explanation of rationale for choice of data collection tool(s), e.g. relevance to the study aims and assessments of tool quality either statistically, e.g. for reliability & validity, or relevant qualitative assessment.
8	Does it have Detailed recruitment data	0	No mention at all	Minimal recruitment data, e.g. no. of questionnaire sent and no. returned.	Some recruitment information but not complete account of the recruitment process, e.g. recruitment figures but no information on strategy used.	Complete data regarding no. approached, no. recruited, attrition data where relevant, method of recruitment.
9	Does it have Statistical assessment of reliability and validity of measurement tool(s) (Quantitative only)	n/a	No mention at all	Reliability and validity of measurement tool(s) discussed, but not statistically assessed.	Some attempt to assess reliability and validity of measurement tool(s) but insufficient, e.g. attempt to establish test–retest reliability is unsuccessful but no action is taken.	Suitable and thorough statistical assessment of reliability and validity of measurement tool(s) with reference to the quality of evidence as a result of the measures used.
10	Does it have Fit between stated research question and method of data collection (Quantitative)	n/a	No research question stated	Method of data collection can only address some aspects of the research question.	Method of data collection can address the research question but there is a more suitable alternative that could have been used or used in addition.	Method of data collection selected is the most suitable approach to attempt answer the research question

11	Does it have Fit between stated research question and format and content of data collection tool e.g. interview schedule (Qualitative	3	No research question stated	Structure and/or content only suitable to address the research question in some aspects or superficially.	Structure & content allows for data to be gathered broadly addressing the stated research question(s) but could benefit from greater detail.	Structure & content allows for detailed data to be gathered around all relevant issues required to address the stated research question(s).
12	Does it have Fit between research question and method of analysis	3	No mention at all	Method of analysis can only address the research question basically or broadly.	Method of analysis can address the research question but there is a more suitable alternative that could have been used or used in addition to offer greater detail.	Method of analysis selected is the most suitable approach to attempt answer the research question in detail, e.g. for qualitative IPA preferable for experiences vs. content analysis to elicit frequency of occurrence of events, etc.
13	Does it have Good justification for analytical method selected	3	No mention at all	Basic explanation for choice of analytical method	Fairly detailed explanation of choice of analytical method	Detailed explanation for choice of analytical method based on nature of research question(s)
14	Does it have Assessment of reliability of analytical process (Qualitative only)	0	No mention at all	More than one researcher involved in the analytical process but no further reliability assessment.	Limited attempt to assess reliability, e.g. reliance on one method.	Use of a range of methods to assess reliability, e.g. triangulation, multiple researchers, varying research backgrounds
15	Does it have Evidence of user involvement in design	3	No mention at all	Use of pilot study but no involvement in planning stages of study design.	Pilot study with feedback from users informing changes to the design.	Explicit consultation with steering group or statement or formal consultation with users in planning of study design.
16	Does it have Strengths and limitations critically discussed	3	No mention at all	Very limited mention of strengths and limitations with omissions of many key issues	Discussion of some of the key strengths and weaknesses of the study but not complete	Discussion of strengths and limitations of all aspects of study including design, measures, procedure, sample & analysis.
	Total score	26/42				
		62%				
